# Spousal Associations between Social Participation and Chronic Diseases among Chinese Middle-aged and Older Adults

**DOI:** 10.1093/geroni/igaf122.3307

**Published:** 2025-12-31

**Authors:** Yunchen Ruan, Mantang Gan

**Affiliations:** Fuzhou University, Fuzhou, Fujian, China (People’s Republic); Fuzhou University, Fuzhou, Fujian, China (People’s Republic)

## Abstract

**Objective:**

While several previous studies have explored the relationship between a spouse’s social participation and the physical health of their partner among Chinese middle-aged and older adults, none have investigated the connection between a spouse’s social participation and chronic diseases in their partner, or the potential gender differences in this relationship. Our study seeks to address this gap.

**Methods:**

This study utilized data from the China Health and Retirement Longitudinal Study (CHARLS) follow-up surveys conducted in 2013, 2015, and 2018. The sample consisted of 3,072 couples aged 45 and older. Chronic diseases were assessed by counting the total number of chronic conditions reported by the participants. Social participation was measured by the number of different types of social activities in which the participants engaged. The actor-partner interdependence model was employed for analysis.

**Results:**

Spousal social participation was significantly associated with the partner’s chronic diseases, with notable gender differences. The wife’s social participation was positively correlated with the husband’s chronic diseases, while the husband’s social participation was negatively correlated with the wife’s chronic diseases. Notably, depressive symptoms played a significant mediating role in the relationship between spousal social participation and the partner’s chronic diseases.

**Conclusions:**

Spouses’ social participation is associated with chronic diseases in their partners among Chinese middle-aged and older adults, with this association being gender-dependent. The government should prioritize building and strengthening social support networks while implementing gender-sensitive measures to promote social participation and improve health outcomes.

